# Ascending aortobifemoral and adjunct carotid bypass grafts

**DOI:** 10.1016/j.jvscit.2023.101203

**Published:** 2023-06-19

**Authors:** Tiago F. Ribeiro, Ricardo Correia, Rita Bento, Nelson Camacho, João Monteiro Castro, Maria Emília Ferreira

**Affiliations:** Serviço de Angiologia e Cirurgia Vascular, Hospital de Santa Marta, Centro Hospitalar Universitário de Lisboa Central, Lisboa, Portugal

**Keywords:** Abdominal aorta, Ascending aorta, Brachiocephalic trunk, Chronic total occlusion

## Abstract

We describe a case of simultaneous ascending aortobifemoral and right common carotid artery bypass to treat a symptomatic brachiocephalic artery and juxtarenal chronic total occlusion in a 68-year-old female patient with unfavorable characteristics for endovascular and standard aortofemoral procedures. Mid-term follow-up revealed sustained remission of symptoms, quality of life quality of life improvement, and patent bypass grafts. In highly selected patients, this solution can be useful when treating other intrathoracic diseases, as well as allowing the simultaneous revascularization of two remote arterial beds.

In patients with disabling vascular claudication, revascularization can improve physical function and quality of life.^1^ With the onset of chronic limb-threatening ischemia, it is the cornerstone for limb preservation.^2^ Endovascular therapy still has limitations in technical success and long-term patency for severely calcified chronic total occlusion (CTO) or juxtarenal lesions. We describe a combination of open surgical procedures to treat a symptomatic brachiocephalic artery (BCA) and juxtarenal CTO using a single aortic inflow source. The patient provided written informed consent for the report of her case details and imaging studies.

## Case report

**Fig 1:**
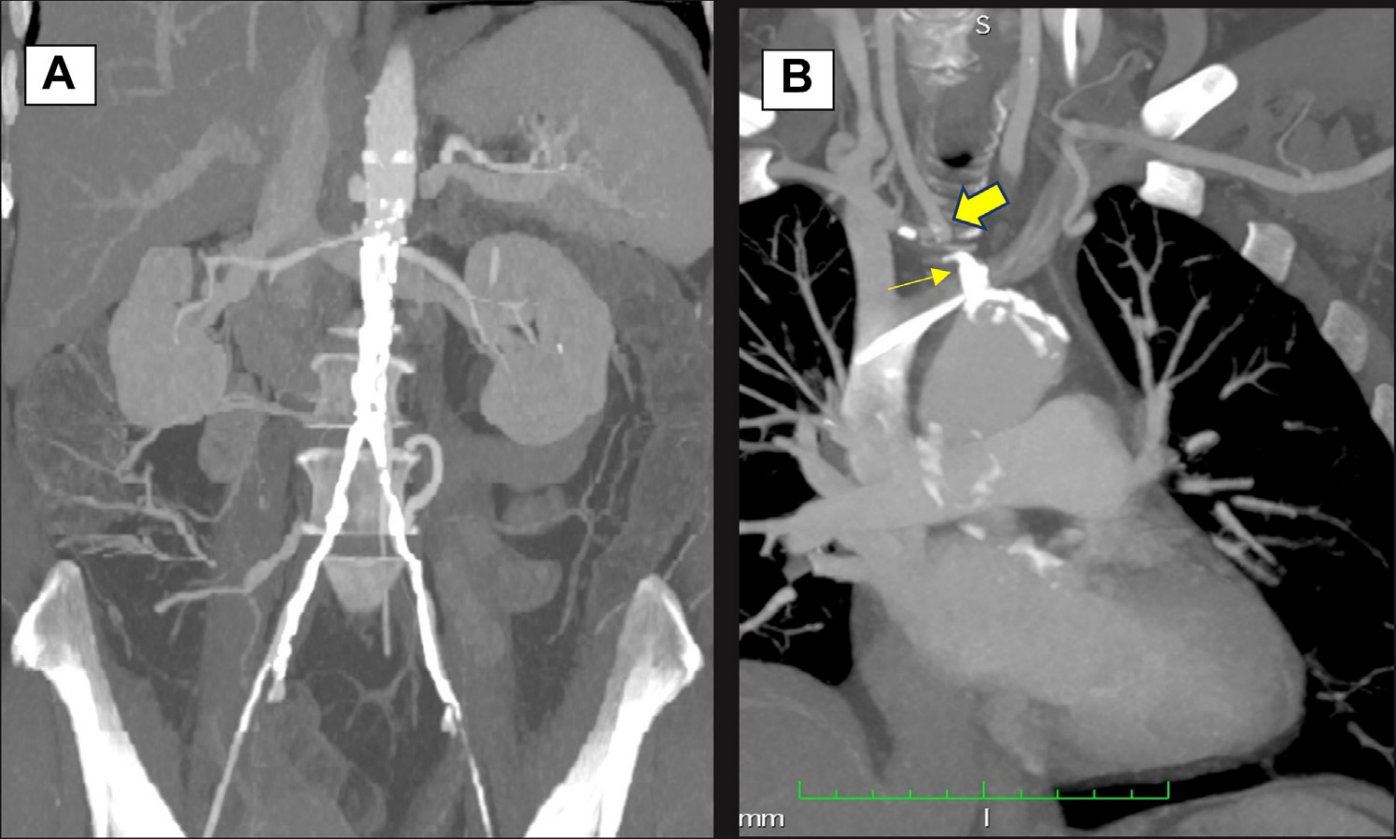
Coronal computed tomography angiographic views showing extensive calcified occlusion of the juxtarenal aorta (**A**) and brachiocephalic artery (BCA; **B**, *narrow arrow*), with a patent carotid artery–subclavian artery bifurcation (*large arrow*).

A 68-year-old woman with a previous smoking habit, hypertension, hyperlipidemia, and obesity (body mass index, 33 kg/m^2^) presented with severe lifestyle-limiting right lower extremity (10 meters) and upper extremity (dominant limb) claudication. The workup revealed a calcified flush BCA occlusion with patency of its bifurcation, calcified left subclavian stenosis (<50%), a juxtarenal calcified aortic occlusion, and bilateral common and superficial femoral artery disease (Fig 1, A and B). She started best medical therapy (ie, clopidogrel 75 mg daily, atorvastatin 40 mg daily, cilostazol 100 mg twice daily), weight loss measures, and a supervised walking program. Despite therapeutic compliance for almost 12 months, her claudication distance worsened; thus, revascularization was considered.

Regarding the revascularization options, the BCA and juxtarenal CTO were considered unlikely to be crossable, with an increased risk of rupture or end-organ compromise during an endovascular procedure. Also, the patient's aortic constraints (ie, pararenal calcification) limited the use of a standard aortobifemoral (ABF) bypass.^3^

We considered simultaneous upper and lower limb revascularization using an ascending aortic inflow source via ascending ABF bypass and BCA bypass. In view of the exposure route, a preoperative cardiopulmonary assessment (including stress echocardiography) was used to exclude any significant pathology. Also, the ascending aortic wall had no significant thrombus or calcification, reducing the risks of aortic side clamping.

**Fig 2:**
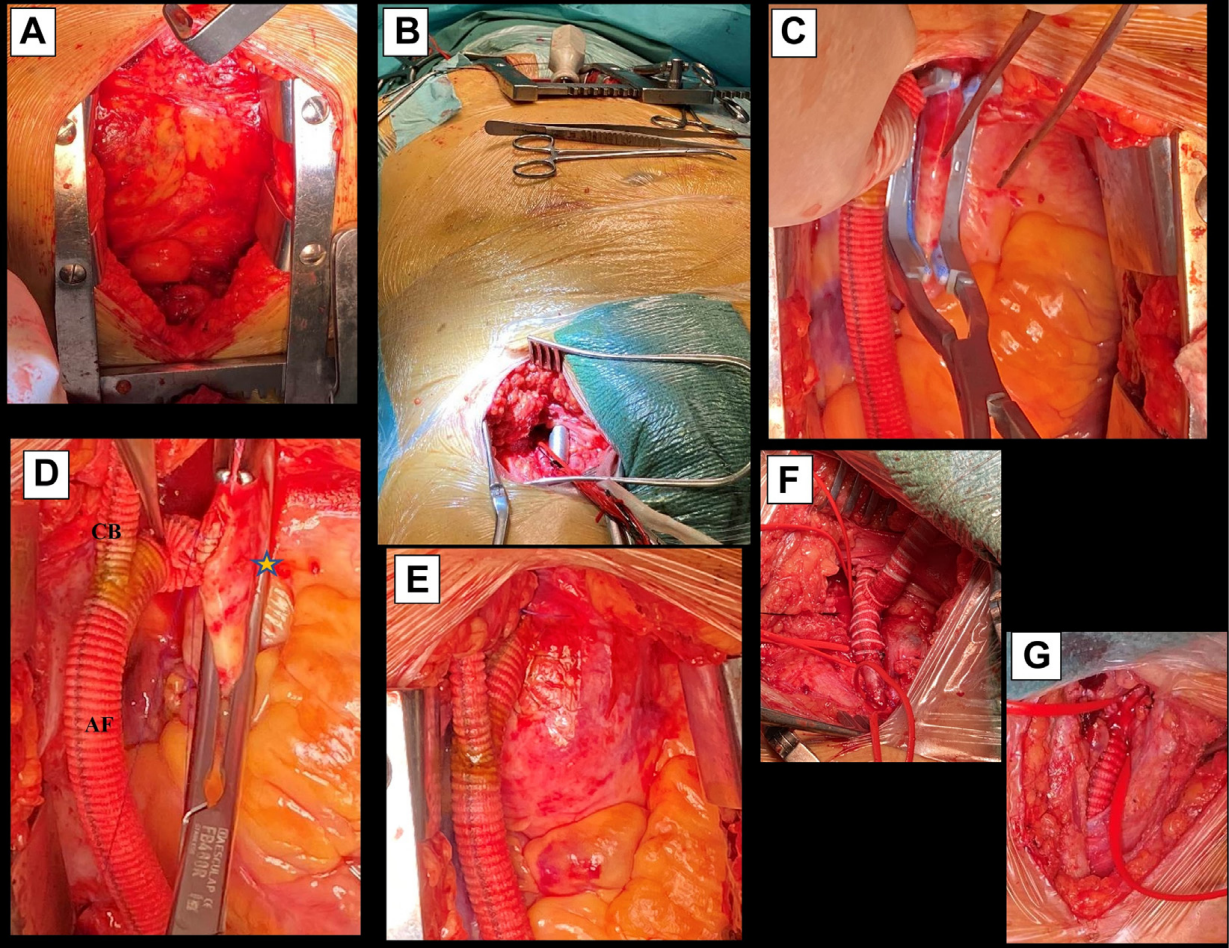
Intraoperative details. **A,** Median sternotomy. **B,** Tunneling through the pericardial cavity and right groin. **C,** Side biting clamping (Satinsky clamp) of the mid-portion of the ascending aorta after graft tunneling. **D,** Prosthetic anastomosis between the right common carotid artery (CCA) graft and bifemoral artery graft and ascending aortic anastomosis (*star*). **E,** Grafts after aortic declamping. **F,** Right groin graft configuration and femoral anastomosis. Note that the aortobifemoral (ABF) graft passes under the inguinal ligament and the premounted crossover on the subcutaneous tissue. **G,** Right CCA anastomosis. *AF,* Ascending aortobifemoral bypass; *CB,* aorto-carotid bypass.

The patient was positioned supine and draped from the neck to both groins. Through a median sternotomy, the ascending aorta and proximal BCA were exposed (Fig 2, A). At this point, a deep coursing and heavily calcified BCA was noted, precluding a safe distal anastomosis and proximal stump ligation. Attention was turned to the right common carotid artery (CCA), which was exposed through a right cervicotomy, followed by tunneling anterior to the innominate vein. Bilateral groin femoral exposure and a suprapubic subcutaneous tunnel connecting both groins were performed. Tunneling between the pericardial cavity and the right groin was then performed. An incision in the anterior diaphragm and posterior rectus sheath connected the pericardial cavity to the abdominal wall. A curved-tip tunneler was then passed anterior to the posterior rectus sheath to the right groin (Fig 2, B). Next, unfractionated heparin (80 IU/kg) was administered.

**Fig 3:**
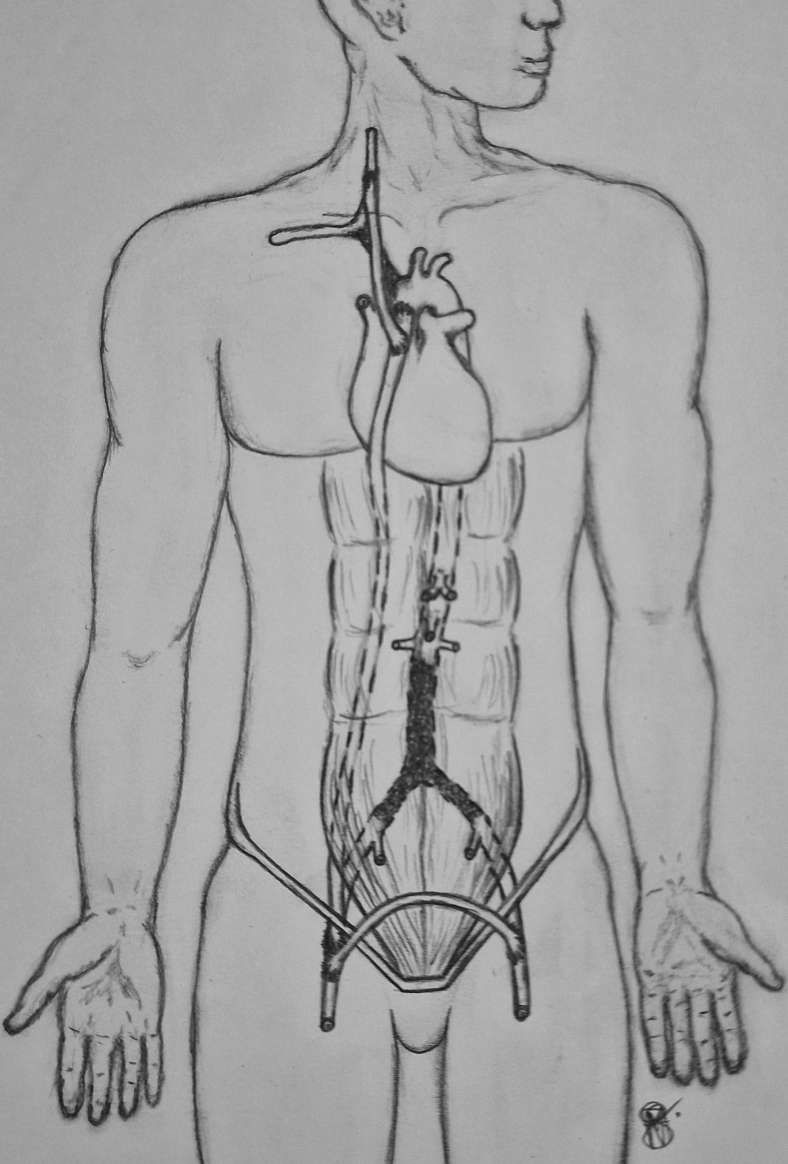
Schematic depiction of the ascending aortobifemoral (ABF) bypass and adjunct right common carotid artery (CCA) graft.

A ringed axillobifemoral 8-mm Dacron graft was chosen for the lower limbs and a nonreinforced 6-mm Dacron tube for the supra-aortic trunk. First, an end-to-side anastomosis between both grafts was performed. Next, the proximal end of the ringed graft was obliquely trimmed, the aorta was partially clamped using a Satinsky clamp), and an aorta–prosthetic end-to-side anastomosis was created (Fig 2, C-E). Subsequently, the graft was passed to the right groin, and the contralateral limb was taken to the left via the suprapubic tunnel. After bilateral femoral endarterectomy, distal end-to-side anastomoses were created (Fig 2, F). Finally, the 6-mm graft was passed through the cervical tunnel, and the carotid artery anastomosis was performed (Fig 2, G). Neuromonitoring was performed using cerebral oximetry. No significant changes during the 190-minute procedure occurred. Retrograde carotid to subclavian arterial flow at the conclusion of the case resulted in a palpable radial pulse (Fig 3).

**Fig 4:**
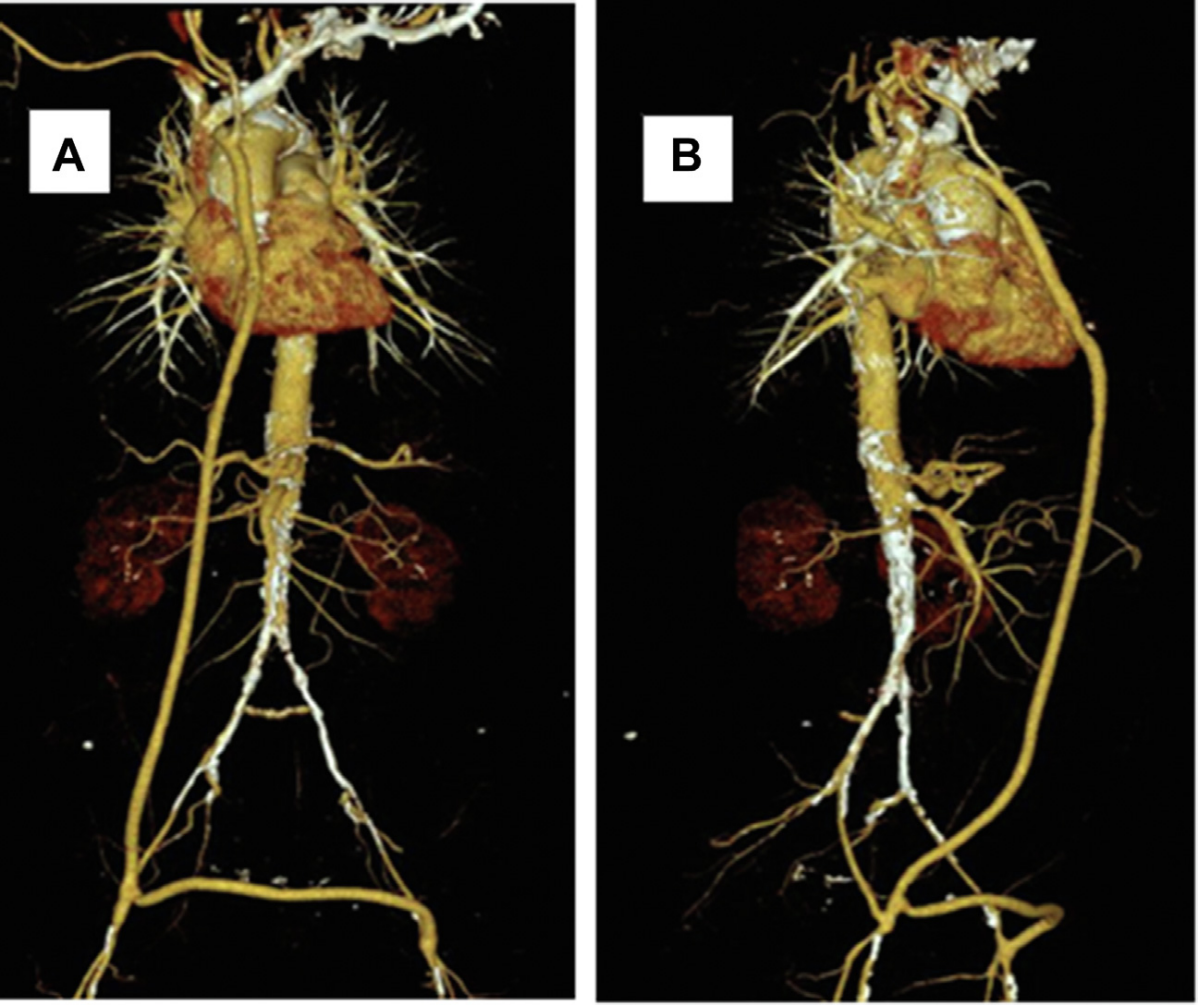
Follow-up computed tomography angiogram at 18 months showing patency of the ascending aortobifemoral (ABF) bypass and carotid artery bypass, without significant stenosis or anastomotic complications.

Her postoperative course was uneventful, and the patient was discharged with bilateral radial, carotid, and femoral artery pulses with long-term clopidogrel and atorvastatin. At mid-term follow-up (18 months), the results were favorable, and the patient was asymptomatic, with significantly improved quality of life and patent bypasses (Fig 4, A and B). Annual ultrasound scans and 5-year computed tomography angiograms are currently planned.

## Discussion

Ascending ABF bypass is not new. Following the initial experience of Frantz et al,^4^ Baird and Madras^5^ performed an ascending aortofemoral bypass by placing the graft ventrally (“ventral aorta”). Despite technical refinements, this has remained the mainstay of this technique.

Our patient presented with severely symptomatic juxtarenal CTO and flush BCA. Some key factors limited the use of endovascular therapy. Her BCA anatomy was associated with a significant risk of dissection, rupture, and/or unintentional side branch coverage. The TASC (TransAtlantic Inter-Society Consensus) class D lesion was considered unlikely to be crossable, and a high risk of aortic rupture or unintentional renal artery coverage or embolization was present. Although the use of stent grafts and/or renal chimneys could limit these risks, their use would have been technically demanding, with uncertain long-term patency.^6^ Thus, an open approach was considered the best overall therapy.

Severe aortoiliac lesions are classically treated with ABF bypass or axillobifemoral bypass. The latter, notwithstanding ongoing patency improvements, was not considered owing to the suboptimal long-term outcomes and the presence of left subclavian stenosis (despite being <50%).^7^ In this setting, ABF bypass remains the gold standard, with 5-year primary patency of 95% reported.^8^ Despite excellent outcomes, certain limitations should be noted, including severe juxtarenal calcification. Regarding BCA disease, bypass in average risk patients usually includes inflow from the ascending aorta, with 5-year primary patency ≤92%.^9^

Considering our patient’s abdominal challenges and the need for concomitant supra-aortic trunk revascularization, a common aortic ascending aortic inflow source was chosen, and ascending ABF bypass with BCA bypass was planned. Although initially used in patients unfit for ABF bypass or axillobifemoral bypass, some groups have reported its use when treating other intrathoracic diseases. Bosse et al^10^ reported their use in eight patients (simultaneous cardiac or supra-aortic procedures), with a 5-year primary patency of 86%. The technique we used was similar; however, for the BCA bypass, there were relevant differences, because they had used a second ascending aortic proximal anastomosis. Bosse et al^10^ performed the proximal supra-aortic graft anastomosis in the proximal part of the ascending aortobifemoral graft, thus minimizing aortic manipulation. The tunneling technique also merits mention. Tunneling anterior to the posterior rectus fascia, taking care to not enter the peritoneal cavity or injure the epigastric vessels, is key. In contrast, a crossover configuration, compared with using another lower abdominal incision to accommodate the graft bifurcation, is arguable. We chose a crossover configuration to reduce the number of incisions and the risk of wound site complications, with, however, unknown long-term patency implications. Also, the graft diameter is debatable, and no “rules” are available regarding this topic. Bosse et al^10^ selected the graft diameter according to the recipient vessel diameter, and Baird et al^11^ routinely used 8- to 10-mm grafts.

Other groups have reported favorable outcomes using the descending thoracic aorta as the inflow source (thoraco-bifemoral bypass).^12^ This technique, however, does not allow for simultaneous BCA revascularization, which would also require a carotid–carotid bypass.

Distal anastomosis in the supra-aortic bypass was performed in the CCA. Classically, BCA bifurcation is selected for the distal anastomosis.^9^ Our decision was technical, because the bifurcation was deep in the upper chest, and its heavy calcification precluded safe proximal stump ligation. Instead, a right subclavian anastomosis to decrease the stroke risk compared with CCA manipulation was considered. However, straightforward exposure and tunneling of an aorto-carotid bypass prevailed. Also, the patient ended with a right radial pulse, without neurologic events. This procedure allowed for single-stage treatment, reducing the operative time and decreasing the risk of laparotomy complications, with maintenance of adequate inflow for both bypasses.

Notwithstanding the low body of evidence, the patency rates seem reasonable.^11^^,^^12^ The major disadvantages are the risk of cerebral embolism due to ascending aortic and CCA manipulation and the limitations regarding future right-sided laparotomy incisions.

## Conclusions

We describe a combination of open surgical techniques to treat a patient with complex vascular issues. Our technique can provide advantages for selected cases, such as simultaneous revascularization of two remote arterial beds, when concomitant heart surgery is needed, and as a last resort for those with abdominal constraints.
